# Effects of supervised structured aerobic exercise training program on fasting blood glucose level, plasma insulin level, glycemic control, and insulin resistance in type 2 diabetes mellitus

**DOI:** 10.12669/pjms.333.12023

**Published:** 2017

**Authors:** Syed Shakil-ur-Rehman, Hossein Karimi, Syed Amir Gillani

**Affiliations:** 1 Syed Shakil-ur-Rehman, PhD Physical Therapy Scholar. Principal/Associate Professor, Riphah College of Rehabilitation Sciences, Riphah International University, Islamabad, Pakistan. University Institute of Physical Therapy, Faculty of Allied Health Sciences, University of Lahore, Lahore, Pakistan; 2 Hossein Karimi, Associate Professor (Emeritus), Adjunct Faculty, University Institute of Physical Therapy, Faculty of Allied Health Sciences, University of Lahore, Lahore, Pakistan; 3 Syed Amir Gillani, Professor and Dean Faculty of Allied Health Sciences, University Institute of Physical Therapy, Faculty of Allied Health Sciences, University of Lahore, Lahore, Pakistan

**Keywords:** type 2 diabetes mellitus, Insulin resistance, glycemic control, plasma insulin, fasting blood glucose level, aerobic exercises

## Abstract

**Objective:**

To determine the effects of supervised structured aerobic exercise training (SSAET) program on fasting blood glucose level (FBGL), plasma insulin level (PIL), glycemic control (GC), and insulin resistance (IR) in type 2 diabetes mellitus (T2DM).

**Methods:**

Riphah Rehabilitation and Research Centre (RRRC) was the clinical setting for this randomized controlled trial, located at Pakistan Railways General Hospital (PRGH), Rawalpindi, Pakistan. Study duration was 18 months from January 1, 2015 to June 30, 2016. Patients of both genders ranging 40-70 years of age with at least one year of history of T2DM were considered eligible according to WHO criteria, while patients with other chronic diseases, history of smoking, regular exercise and diet plan were excluded. Cohorts of 195 patients were screened out of whom 120 fulfilled the inclusion criteria. Amongst them 102 agreed to participate and were assigned to experimental (n=51) and control (n=51) groups. Experimental group underwent SSAET program, routine medication and dietary plan, whereas the control group received routine medication and dietary plan, while both group received treatment for 25 weeks. The blood samples were taken at baseline and on the completion of 25 weeks. The investigation of fasting blood glucose level, plasma insulin level, and glycemic control was conducted to calculate IR.

**Results:**

Patients with T2DM in experimental group (n=51) treated with SSAET program, routine medication and dietary plan significantly improved FBGL (pre-mean= 276.41±25.31, post-mean=250.07±28.23), PIL (pre-mean=13.66±5.31, post-mean=8.91±3.83), GC (pre-mean=8.31±1.79, post-mean 7.28±1.43), and IR (pre-mean=64.95±27.26, post-mean 37.97±15.58), as compared with patients in control group treated with routine medication and dietary plan in whom deteriorations were noted in FBGL (pre-mean=268.19±22.48, post-mean=281.41±31.30), PIL(pre-mean=14.14±5.48, post-mean=14.85±5.27) GC (pre-mean=8.15±1.74, post-mean=8.20±1.44, and IR (pre-mean=64.49±23.63. post-mean=70.79 ±23.30). Statistically at the baseline the results were not significant (p>0.05), but at the completion of 25 weeks intervention all the variable showed significant results (p<0.05.

**Conclusion:**

It is concluded that a 25 weeks SSAET program along with routine medical management is more effective treatment in the management of fasting blood glucose level, glycemic control, plasma insulin level and insulin resistance as compared with routine medical management and dietary plan in the management of T2DM.

## INTRODUCTION

According to American Diabetic Association (ADA) blood glucose level higher than normal (100-125 mg/dl) is considered as diabetes mellitus (DM). in type 2 DM the body does not use insulin properly leading to higher level of fasting blood glucose, increased plasma insulin level, impaired glycemic control (higher HbA1c) and insulin resistance (IR). IR is a pathological state where the human body cells in muscles fail to respond appropriately to the hormone insulin (sensitivity).

IR often remains undetectable, but it produces impaired glucose tolerance (IGT) or T2DM, leading to systemic complications. Clinically it is known as IR syndrome and it produces multi-systemic signs and symptoms including, high blood sugar, loss of concentration, sleepiness, intestinal bloating, weight gain, elevated triglycerides level, hypertension, and depression.^1^ Risk factors associated for IR are genetic predisposition, age 40-50 or older, central obesity, physical inactivity, hypertension, hypertriglyceridemia, low HDL level and previous diabetes. Certain pathologies like obesity, hyperlipidemia, hypertension, infections like hepatitis C and certain liver pathologies also contribute along with steroids and other medications to the occurrence of IR.^2^

IR is directly related to β-cell function and its decreased insulin secretory response and inability to overcome the state of chronic hyperinsulinemia. High resistance to insulin-stimulated glucose uptake and hyperinsulinemia increase the possibility of three major interrelated diseases including T2DM, hypertension and coronary artery disease (CAD).^3^ Obesity is one of the key risk factors which increase the possibility of IR and T2DM due to increased secretions of non-esterified fatty acids, glycerol, hormones, pro-inflammatory cytokines and other factors from the adipose tissues in obese person’s body. Increased IR causes dysfunction of pancreatic islet -cells, which leads to uncontrolled level of blood glucose, and it is considered a great risk factor in the development of T2DM in obese individuals.^4^

Physical inactivity is highly associated with the development of insulin resistance, dyslipidemia, high blood pressure, and impaired micro-vascular function in healthy population. The long and short term physical inactivity causes metabolic and vascular changes, which lead towards IR and ultimately to T2DM.^5^ A vigorous and non-vigorous physical activity programs produced positive impacts on insulin sensitivity and reduction in IR in mild to moderate type of T2DM patients and supports the recommendation of moderate physical activity in patients with impaired glucose tolerance and T2DM.^6^ Ageing has no correlation with the development of IR, while physical inactivity and obesity has a direct relation with IR occurrence.^7^

Managing certain modifiable factors such as diet, exercise, smoking and stress are thought to contribute to the control of IR. Lifestyle intervention to address these factors appears to be a valuable part of any therapeutic approach.^8^ A positive impact of mild to moderate exercise intervention on IR in young patients has been reported.^9^ The current study (*Trial No:* (Trial ID ISRCTN16466697) was designed to determine the effects of a 25 weeks supervised structured aerobic exercise training (SSAET) program on fasting blood glucose level (FBGL), plasma insulin level (PIL), glycemic control (GC), and insulin resistance (IR) in type 2 diabetes mellitus (T2DM).

## METHODS

Riphah Rehabilitation and Research Centre (RRRC) located at Pakistan Railways General Hospital (PRGH), Rawalpindi, Pakistan was the clinical setting for the current randomized controlled trial. The total duration of the study was the period of 18 months from January 1, 2015 to June 30, 2016.

Male and female participants aging 40-70 years with at least one year history of T2DM, fulfilling the WHO criteria were considered eligible, while patients with the history of chronic systemic diseases, smoking, regular exercise and diet plan were excluded. A cohort of 195 patients was screened out as per the inclusion criteria. Of them 120 fulfilled the inclusion criteria. Finally 102 of them agreed for participation and then randomly assigned to experimental (n=51) and control (n=51) groups.

Experimental group underwent SSAET program, routine medication and dietary plan, whereas the control group received routine medication and dietary plan for 25 weeks at the rate of three days per week. The blood samples were taken on baseline and at the completion of 25 weeks. the investigation of fasting blood glucose level, plasma insulin level, and glycaemic control was conducted to calculate IR.

Fasting blood glucose level was recorded directly by glucometer. Ethylenediaminetetraacetic acid (EDTA) blood sample (2ml in purple tube) was taken for glycemic control (HbA1c) and HbA1c kit of Tianjin MD Pacific Technology Company of China was used. The blood sample (2ml in yellow tube) was taken centrifuged for 10 minutes and serum was separated for plasma insulin level by using microlisa human insulin kit made by Amgenix USA. IR was calculated by homeostatic Model Assessment of Insulin Resistance (HOMA-IR) by using the formula: IR= (insulin mU/L x glucose mg/dl) / 405. All the laboratory investigations were done at postgraduate research lab at Islamic International medical college, Riphah International University, Rawalpindi, Pakistan.

Medically graded treadmill was used in SSAET program along with telemetric monitoring of heart rate, blood pressure, oxygen saturation and ECG. The 25 week SSAET program was divided into five phases of five weeks each. In phase-1, the duration of single session was 10 minutes and the total duration per week was 30 minutes. Thirty minutes increase per week was followed in the subsequent 4 phases. Normal speed of each participant was used as a treadmill speed and was determined by 20 meter distance test at baseline. Phase-1 was conducted at zero degree inclination with the ground, phase-2 at 3 degree, while 3 degree increase was followed in the subsequent three phases. Pre and post intervention statistical data analysis was done using SPSS software version- 20 and independent t test was applied at 95% level of significance.

## RESULTS

The sample size was 102 with mean age of 54.73 ± 8.17 years, where mean age of the experimental group was 53.74 ± 8.75 and the mean age of the control group B was 55.08 ± 7.67 years. Sixty eight (66.7%) patients were female and 34 (33.3%) were male, where majority (97.05%) of participants were married and only 3 (2.94%) were single. Mean years with T2DM after diagnosis were 7.12 years, with minimum of one year and maximum of 16 years. The total 38 (37.25%) participants had job and 64 (62.74%) were jobless/ retired. Past history of smoking was positive for 19 (18.62%) individuals and majority 83 (83.37%) demonstrated no history of smoking.

Clinically the experimental group (n=51) treated with SSAET program, routine medication and dietary plan improved significantly in FBGL (pre-mean= 276.41±25.31, post-mean=250.07±28.23), PIL (pre-mean=13.66±5.31, post-mean=8.91±3.83), GC (pre-mean=8.31±1.79, post-mean 7.28±1.43), and IR (pre-mean =64.95 ±27.26, post-mean 37.97±15.58), as compared with patients in control group treated with routine medication and dietary plan in whom deteriorations were noted in FBGL (pre-mean = 268.19 ±22.48, post-mean=281.41±31.30), PIL(pre-mean=14.14±5.48, post-mean=14.85±5.27) GC (pre-mean=8.15±1.74, post-mean=8.20±1.44, and IR (pre-mean=64.49±23.63. post-mean = 70.79 ±23.30).

Statistically the experimental group showed significantly better improvements than control group in managing FBGL, plasma insulin level PIL, GC, and IR in T2DM. Detailed comparison of mean, standard deviation and p-value regarding the experimental and control groups for FBGL, plasma insulin level PIL, GC, and IR in T2DM are shown in Table-I.

**Table-I:** Showing comparison of mean, standard deviation and p-value in experimental and control groups for fasting blood glucose level (FBGL), plasma insulin level (PIL), glycemic control (GC), and insulin resistance (IR) in T2DM.

*Variables*	*Pre mean ±SD Exp group (n=51)*	*Pre mean ±SD Control group (n=51)*	*p-value*	*Post mean ±SD Exp group (n=51)*	*Post mean ±SD Control group (n=51)*	*p-value*
Fasting Blood Glucose Level	276.41 ± 25.31	268.19 ± 22.48	0.086	250.07 ± 28.23	281.41 ± 31.30	0.001
Plasma Insulin Level	13.66 ± 5.31	14.14 ± 5.48	0.652	8.91 ±3.83	14.85 ± 5.27	0.001
Glycemic Control	8.31 ±1.79	8.15 ± 1.74	0.643	7.28 ± 1.43	8.20 ± 1.44	0.002
Insulin Resistance	64.95 ± 27.26	64.49 ± 23.63	0.927	37.97 ± 15.58	70.79 ± 23.30	0.001

## DISCUSSION

Results of the current study showed that 51 patients with T2DM in experimental group who were treated three times a week for 25 weeks with SSAET program, routine medication and dietary plan produced significant improvements in FBGL, PIL, GC and IR as compared with the other 51 patients in control group treated for the same duration of time with routine medication and dietary plan.

Avery and Walker (2001) evaluated the effect of a single session cycling of 30 minutes duration on blood glucose and insulin level in population with gestational diabetes mellitus (GDM) of age ranged 18-38. They reported significant reduction in blood glucose level after exercise as compared with the baseline.^10^ Our results also showed marked reduction in FBGL among patients group treated with SSAET program, routine medication and dietary plan.

Thompson and colleagues (2001) investigated the acute versus chronic effects of a single session exercise equal to 40% of maximal capacity. They reported both acute and chronic effects of exercise on insulin sensitivity, glucose homeostasis, and blood pressure and blood lipids, but the intensity, duration, and energy expenditure required to produce these effects are not clearly defined in the literature.^11^ The current study also showed improvements in FBGL, PIL, and glycemic control in experimental (exercise) group.

Adams (2013) carried out a meta-analysis on the impact of brief high-intensity exercise on blood glucose levels and foundsix studies of non-diabetics (51 males, 14 females) performing 7.5 to 20 minutes/week of high intensity exercise were reviewed. The six studies that were reviewed suggested that sprint interval training of two weeks duration increased insulin sensitivity up to three days. Maximal interval running of two weeks with total duration of 40 minutes per week positively influenced blood glucose level. The same effects are also produced by running with 65% VO_2max_ for a duration of 150 minutes/week. In addition to that it was also suggested that a single exercise session of 44 seconds to 13 minutes of high intensity exercise also improved blood glucose level in patients with T2DM.^12^ The present study also demonstrated positive effects on PIL in exercise (experimental) group.

Another meta-analysis conducted by Boule and team (2001) to review the effects of exercise on glycosylated hemoglobin (HbA_1c_) and body mass in patients with Type 2 diabetes. They concluded that Exercise improves glycemic control by reducing HbA1c along with decrease risk of diabetic complications, while no changes were noted in Body Mass Index (BMI) when the exercise groups was compared with the control groups.^13^ Carmen and colleagues (2002) conducted an RCT and tested a progressive resistance exercise of 16 weeks at three days per week on glycemic control and glycogen storage in muscles. They reported improvements in glycemic control from 8.7 ± 0.3 to 7.6 ± 0.2%, increased glycogen storage in muscles from 60.3 ± 3.9 to 79.1 ± 5.0 mmol glucose/kg muscle and minimize amount of medications used by 72% as compared with the patients in control group (non-exercise group).^14^ Results of our study also presented improvements in HbA1c score in the group of patients treated with SSAET program, routine medication and dietary plan.

Kiens (2006) conducted another research study on Skeletal Muscle Lipid Metabolism in Exercise and Insulin Resistance and reported a positive impact of exercises on reducing VLDL as well as Intra Muscular Tri Glyceride (IMTG). These two lipids act as fuel during exercises. In male subjects the contribution of IMTG was low covering 10% of the energy provision during fasting exercises. In female subjects the IMTG met a large proportion of energy requirement. Thus contributed positively in reduction of adipose tissue and leads to decrease insulin resistance.^15^

Bollinger and team (2011) conducted a review study on exercise and insulin resistance. They concluded that exercise has the potential to treat IR by improving insulin sensitivity, increased oxygen uptake capabilities of skeletal muscles, and enhanced beta cell function along with modifications in sign, symptoms and risk associated with IR.^16^

Ivy and associates (1997) concluded that exercises play an important role in fat reduction from central regions of the human body and significantly contribute to improvements in insulin sensitivity. Exercise training also stimulates muscle development and prevents muscle atrophy. Several months of exercise significantly increase muscle response to glucose uptake without affecting glucose tolerance.^17^ Improvements in IR also resulted in our study by experimental group along with FBGL, PIL, and GC.

As mentioned, mild deterioration was noted in FBGL PIL, GC and IR in control (no-exercise) group, treated with routine medication and dietary plan. Barbara (2000) reported association between obesity, IR and type 2 diabetes mellitus and exercise improves obesity and physical inactivity may elicit weight gain.^18^ Richter and group (1982) presented in their study that exercise improves insulin sensitivity in muscles.^19^

## CONCLUSION

It is concluded that a 25 weeks SSAET program along with routine medical management is more effective treatment in the management of fasting blood glucose level, glycemic control, plasma insulin level and insulin resistance as compared with routine medical management and dietary plan in the management of T2DM.

### Authors` Contribution

**SSR & HK**: Conceived, designed and did statistical analysis & editing of manuscript.

**SSR, HK& SAG**: Did data collection and manuscript writing.

**HK** approved the final version of the manuscript.

**SSR:** Takes the responsibility and is accountable for all aspects of the work in ensuring that questions related to the accuracy or integrity of any part of the work are appropriately investigated and resolved.
